# Use of olanzapine to treat agitation in traumatic brain injury: study protocol for a randomised controlled trial

**DOI:** 10.1186/s13063-020-04553-2

**Published:** 2020-07-20

**Authors:** Ruby K Phyland, Adam McKay, John Olver, Mark Walterfang, Malcolm Hopwood, Amelia J Hicks, Duncan Mortimer, Jennie L Ponsford

**Affiliations:** 1 Monash Epworth Rehabilitation Research Centre, 185-187 Hoddle Street, Richmond, Victoria 3121 Australia; 2 School of Psychological Sciences, Monash University, 18 Innovation Walk, Clayton Campus, Wellington Road, Clayton, Victoria 3800 Australia; 3 Turner Institute for Brain and Mental Health, Monash University, Level 5, 18 Innovation Walk, Clayton Campus, Clayton, Victoria 3800 Australia; 4 Department of Psychology, Epworth HealthCare, 29 Erin Street, Richmond, Victoria 3121 Australia; 5 Rehabilitation Medicine, Epworth HealthCare, 89 Bridge Rd, Richmond, Victoria 3121 Australia; 6 Department of Psychiatry, University of Melbourne, Royal Melbourne Hospital, Level 1 North Block, Grattan Street, Parkville, Victoria 3052 Australia; 7 Royal Melbourne Hospital, 300 Grattan St, Parkville, Victoria 3050 Australia; 8 Florey Institute of Neuroscience and Mental Health, University of Melbourne, 30 Royal Parade, Parkville, Victoria 3052 Australia; 9 Albert Road Clinic Professorial Psychiatry Unit, University of Melbourne, 31 Albert Rd, Melbourne, Victoria 3004 Australia; 10 Centre for Health Economics, Monash Business School, Monash University, Building H, Level 5, Caulfield Campus, Clayton, Victoria 3145 Australia

**Keywords:** Traumatic brain injury, Agitation, Post-traumatic amnesia, Pharmacological intervention, Olanzapine, Antipsychotic, Randomised controlled trial

## Abstract

**Background:**

Agitation is common in the early stages of recovery from traumatic brain injury (TBI), when patients are in post-traumatic amnesia (PTA). Agitation is associated with risk of harm to patients and caregivers. Recent guidelines recommend that agitation during PTA is managed using environmental modifications. Agitation is also frequently treated pharmacologically, with the use of atypical antipsychotics such as olanzapine among the most common. This is despite a lack of well-designed studies to support the use of antipsychotics within this context. This study will be a double-blind, placebo-controlled randomised controlled trial. We will examine the efficacy, safety, cost-effectiveness and outcomes associated with the use of olanzapine for reducing agitation in patients in PTA following TBI over and above recommended environmental management.

**Methods:**

Fifty-eight TBI rehabilitation inpatients who are in PTA and are agitated will receive olanzapine or placebo for the duration of PTA. All participants will additionally receive optimal environmental management for agitation. Measures of agitation, PTA and health will be undertaken at baseline. Treatment administration will begin at a dose of 5 mg daily and may be escalated to a maximum dose of 20 mg per day. Throughout the treatment period, agitation and PTA will be measured daily, and adverse events monitored weekly. Efficacy will be assessed by treatment group comparison of average Agitated Behaviour Scale scores during PTA. Participants will cease treatment upon emergence from PTA. Agitation levels will continue to be monitored for a further 2 weeks, post-treatment measures of health will be undertaken and cognitive and functional status will be assessed. Level of agitation and functional health will be assessed at hospital discharge. At 3 months post-discharge, functional outcomes and health service utilisation will be measured.

**Discussion:**

This trial will provide crucial evidence to inform the management of agitation in patients in PTA following TBI. It will provide guidance as to whether olanzapine reduces agitation over and above recommended environmental management or conversely whether it increases or prolongs agitation and PTA, increases length of inpatient hospitalisation and impacts longer term cognitive and functional outcomes. It will also speak to the safety and cost-effectiveness of olanzapine use in this population.

**Trial registration:**

ANZCTR ACTRN12619000284167. Registered on 25 February 2019

## Background

The Australian Institute of Health and Welfare has reported a national traumatic brain injury (TBI) incidence of approximately 107 per 100,000 population per annum [1]. Productivity loss, economic costs and ongoing disability associated with TBI render this health condition a significant global public health concern [1–3]. Traumatic brain injury is frequently followed firstly by a period of coma and secondly by a period of post-traumatic amnesia (PTA). In moderate to severe TBI, PTA represents a period of recovery spanning days to months wherein the patient experiences generalised cognitive disturbance. This cognitive state is characterised by anterograde and retrograde amnesia, confusion, disorientation, attentional dysfunction, low arousal and poor awareness [4–7]. Patients in PTA often exhibit severe neurobehavioural symptoms [8–10]. Perhaps the most challenging of these is agitation, defined as an excess of behaviour involving restlessness, disinhibition and emotional lability [11–13]. When using a standardised measure of agitation, approximately one third of individuals exhibit agitation during the early stages of recovery from TBI [11, 14]. Agitation is problematic for patients, families and healthcare workers. Restlessness may lead to falls, and aggressive behaviour may result in self-harm, destruction of property and violence towards others [15, 16]. Agitation is associated with poorer rehabilitation engagement and longer hospital stays, and agitated patients reportedly exhibit poorer functional outcomes and less frequent return to private homes [8, 14, 17, 18].

Agitation may be managed using environmental modifications or pharmacological intervention. However, there is a clear lack of evidence to underpin the use of either of these approaches. A recent systematic review concluded that there exists a paucity of well-designed, adequately powered and controlled studies to support the use of pharmacological intervention for neurobehavioural symptoms during PTA, that clear evidence of efficacy for any pharmacological intervention is lacking and that no pharmacological treatment recommendations could be made [19]. This sentiment is echoed throughout the literature [7, 20–24]. Despite a lack of evidence supporting the efficacy and safety of any pharmacological intervention for agitation during PTA, the use of antipsychotics and anticonvulsants is common [12, 25, 26]. The use of newer atypical antipsychotics is preferred over older typical agents due to findings suggesting more favourable cognitive and motor outcomes associated with their use [27–30]. Such evidence is scant, and questions remain as to whether these agents are effective, and whether their use may have negative side effects such as increasing confusion, prolonging PTA duration and length of hospital stay and ultimately impairing recovery [27–35]. Further, it has been theorised that the administration of antipsychotic agents in TBI patients may impair arousal and cognition, exacerbating confusion, disorientation and drowsiness and in turn deepening PTA [7, 36]. Impairments in cognitive performance following TBI have been reported in animal models administered haloperidol [27–29, 35] and risperidone [27, 28, 35] but not olanzapine [29]. Additionally, antipsychotic administration was associated with prolonged PTA duration in several case series [32, 33, 37]. As improvement in cognition is thought to precede the resolution of agitation [5, 6, 26], the sedating effects of neuroleptics could potentially prolong PTA and serve to heighten or prolong agitation. In the face of such scant evidence, physicians are currently forced to rely on clinical experience, expert advice, low-level evidence and empirical assumptions of how similar conditions are managed in order to make treatment decisions, possibly risking poorer outcomes for patients and exposing staff and caregivers to risk of harm.

Olanzapine does not show demonstrated effects on cognitive function or recovery following TBI [29] and has a favourable safety profile when used to treat agitation in other conditions [38–41]. It is therefore arguable that olanzapine is suitable for treating agitation in the acute stages following TBI. Olanzapine is commonly prescribed during PTA [26]. There is however a complete lack of quality data regarding the efficacy, harms and other outcomes associated with the use of this drug for the treatment of agitation during the PTA period [19]. We have therefore chosen olanzapine as the active intervention for this placebo-controlled randomised controlled trial. Current evidence for the use of antipsychotics for the management of neurobehavioural symptoms during PTA is limited to case series with no formal comparison condition [19, 32, 42, 43]. Selection of a placebo comparator minimises bias and allows for collection of a higher level of evidence than that presently available. All study participants will receive optimal environmental management for agitation as per guidelines [7] and may receive a ‘rescue dose’ of olanzapine should agitation escalate to unmanageable levels. This permits the evaluation of olanzapine efficacy in conjunction with optimal environmental management over and above environmental management alone. Additionally, optimal environmental management allows for the ethical provision of medical care to this population in line with current guidelines [7] and the reduction of potential risks associated with agitation. The choice of a placebo-controlled design is therefore considered both scientifically rigorous and ethical.

The primary aim of this trial is to evaluate the efficacy of the atypical antipsychotic olanzapine in reducing agitation in patients in PTA following TBI over and above recommended environmental management. We hypothesise that patients who are administered olanzapine will show lower levels of agitation during PTA than those randomised to a placebo control group receiving optimal environmental management. The secondary aims of this research are to determine (1) the safety of olanzapine administration in PTA patients, whether olanzapine treatment: (2) reduces the number of clinically agitated days during PTA, (3) alters level of agitation at hospital discharge, (4) alters level of cognition in PTA, (5) alters PTA duration, (6) alters cognitive performance following PTA emergence, (7) alters length of inpatient hospital stay and (8) alters functional independence at discharge and 3-month follow-up. Finally, this research aims to evaluate the cost-effectiveness of olanzapine administration in PTA patients.

## Methods/design

This study will examine the efficacy of the atypical antipsychotic olanzapine in reducing agitation in patients in PTA following TBI. We will employ a parallel-group, double-blind, placebo-controlled, two-arm superiority randomised controlled trial with 1:1 allocation ratio. Participants will be 58 patients with TBI in PTA and experiencing clinically significant agitation recruited from the Acquired Brain Injury (ABI) Rehabilitation Unit at Epworth HealthCare, Richmond, Victoria, Australia. This private, not-for-profit urban hospital treats a significant proportion of patients with severe TBI in Victoria, Australia.

### Inclusion criteria

(1) Currently under hospital care at Epworth HealthCare for TBI rehabilitation, (2) a history of blunt head trauma with a loss of consciousness and an initial Glasgow Coma Scale of 3–14 and/or a period of PTA, (3) judged to be in PTA (score < 12) on the Westmead Post Traumatic Amnesia Scale (WPTAS; [44, 45]), (4) judged to be in a state of clinically significant agitation (score > 21) on the Agitated Behaviour Scale, (5) aged 18 years or older, (6) provision of signed and dated Medical Treatment Decision Maker Participant Information and Consent Form by the participant’s Medical Treatment Decision Maker (MTDM; during PTA) and (7) provision of signed and dated Participant Information and Continuing Consent Form by the participant (upon PTA emergence).

### Exclusion criteria

(1) History of premorbid neurocognitive decline (e.g. dementia, stroke); (2) currently requiring antipsychotic treatment for psychotic illness; (3) current use of medications for agitation, including olanzapine, from which they are unable to be weaned; (4) pregnancy or lactation; (5) known allergy to olanzapine; (6) participation in another study involving an investigational product; (7) otherwise determined to be unfit for participation due to medical considerations.

### Recruitment

Patients admitted to the Epworth HealthCare ABI Unit are routinely screened for PTA status using the WPTAS on admission. Those deemed to be in PTA have the WPTAS and ABS completed on a daily basis by a clinical neuropsychologist or nurse until they emerge from PTA. Study staff will be alerted to potential participants by ABI ward staff. Potential participants include patients in PTA who are exhibiting clinically significant agitation or thought likely to become agitated (due to factors such as agitation during acute hospitalisation). Due to their confusion and inability to lay down new memories, patients in PTA are not cognitively capable of consent. Recruitment will therefore target the patient’s MTDM. This will follow a two-stage process. Firstly, the study will be introduced to the patient’s MTDM by a member of their treating medical team and verbal consent for contact from study staff will be gained. Rapid consent processes are important to recruitment given the time-limited nature of PTA. The decision to introduce the trial to MTDMs of those patients determined likely to become agitated (rather than only those presently agitated) has been made in an effort to expedite the recruitment process and avoid exclusion of eligible participants. Agitation levels of those patients not presently agitated are monitored by study staff. If consent for contact is obtained and the patient is agitated at any stage during PTA, study staff will then contact the MTDM to carry out study consent procedures.

Participant recruitment commenced in June 2019, with the aim of recruiting participants until June 2022. Given that the full study protocol will have a duration of approximately 3 months, on average, data collection will continue until the end of September 2022. Epworth HealthCare treats more than 110 new patients with TBI annually, of whom 80 are likely to be in PTA on admission. Recent research studying this population within the Epworth HealthCare ABI ward [26] showed that 42.4% of TBI patients exhibited significant agitation according to the ABS whilst in PTA. Therefore, approximately 34 patients in PTA are likely to exhibit clinically significant agitation annually. Allowing for declined consent and ineligibility, we envisage recruitment of our sample over a 41-month period.

Participants for whom consent is obtained and who are randomised but do not receive the study intervention (e.g. due to the expiration of agitation) will not be replaced. Participants for whom consent is obtained but who are not randomised will be replaced. Participants for whom consent is obtained and who are randomised, receive the study intervention and are subsequently withdrawn from participation will not be replaced. Participants and MTDMs will not be paid for their participation in the trial.

### Consent

#### Identifying an Advance Care Directive/Medical Treatment Decision Maker

Due to their confusion and inability to lay down new memories, it is not possible to obtain consent from patients in PTA. In such circumstances, Australian law stipulates that researchers must first identify the presence of an Advance Care Directive (ACD; a legal document describing the patient’s wishes regarding their medical care). If there is a relevant instructional directive refusing a medical procedure involved in the research project, dissent will be assumed. If there is no instructional directive relevant to one or more of the study’s procedures, consent to research must be made by the patient’s MTDM, the individual who has legal authority to make medical decisions about the patient. If no ACD or MTDM can be identified for a possible participant, that patient’s enrolment into the study will be sought from the Office of the Public Advocate, Victoria, Australia’s governing body who may make such decisions according to law. Identification of an ACD/MTDM will be documented.

#### MTDM consent

If no relevant instructional directive refusing a medical procedure involved in the trial exists and the patient’s MTDM verbally consents to contact, consent will be sought by study staff in-person or via telephone. The option of telephone consent from study staff is being used given the challenges of gaining in-person access to the MTDM in an inpatient hospital setting before the expiration of agitation and PTA. Telephone consent will be thoroughly documented and include detailed verbal explanation of the study and opportunity to have questions answered. If telephone consent is obtained, this will be followed by written consent in-person or via post. If telephone consent is obtained but signed consent is not returned via post, telephone consent and documented identification of that individual as the MTDM will be considered sufficient.

#### Participant consent

Participant consent will be sought by study staff following that participant’s emergence from PTA and having been declared cognitively capable of consent by their treating clinical neuropsychologist. Failure to meet either of these criteria will result in the consent of the MTDM being maintained. In such cases, study staff will continue to monitor whether participants meet criteria for consent for the duration of their admission to the Epworth HealthCare ABI ward.

#### General aspects

All written consent procedures will employ a Medical Treatment Decision Maker Participant Information and Consent Form or Participant Information and Continuing Consent Form, as appropriate. In-person consent will include verbal explanation of the study and the opportunity to have any questions answered. In all instances, two copies of the consent form will be signed by the consenting party and study staff. Each party will retain one copy.

Some physicians and clinical neuropsychologists involved in an individual’s treatment whilst at the Epworth HealthCare ABI Unit are investigators on this study. In an attempt to mitigate any influence this dual relationship may have on the decision of a patient or MTDM to participate in this research, consent procedures will be carried out by a study staff member who is not a part of the patient’s treating medical team. Further, patients and MTDM will be assured that refusal to participate will not impact upon the standard of treatment offered by Epworth HealthCare.

Every effort will be made to enrol participants from non-English speaking backgrounds into the study, provided sufficient communication can be established in order for consent to be obtained, and study procedures are both understood and able to be completed. In the case of reading or writing difficulties, study staff may read the consent form aloud and/or obtain verbal consent in the presence of a witness. Both the witness and researcher obtaining verbal consent will sign and date the applicable consent form to document consent.

Participants and MTDMs may withdraw consent at any time. Should consent be withdrawn, permission will be sought to use data collected up until the point of withdrawal, and for the continued collection of data that does not require active participation by the participant (that is, measures that the hospital would undertake regardless of study participation). The participant’s or MTDM’s wishes regarding this matter will be reflected on the Form for Withdrawal of Participation, found at the end of the appropriate consent form.

This trial is not affiliated with any ancillary studies. Hence, consent to such research will not be sought.

### Screening

Study staff will be alerted to potential participants (in PTA according to the WPTAS and exhibiting or thought likely to exhibit agitation) by Epworth HealthCare ABI ward staff. Following consent procedures, a member of study staff or ABI unit medical registrar will screen this patient for study eligibility. Following confirmation of eligibility, the participant will be allocated the next available sequential participant number.

Some patients may already be taking medications for agitation, such as olanzapine, at this time. If they are deemed by study psychiatrists and the participant’s treating physician as able to be weaned off these medications, this will be done. Commencement of the study product may be carried out as participants are weaned off existing agitation medication, should this be deemed safe by study psychiatrists and the participant’s treating physician. In regard to current olanzapine use, commencement of study product may be initiated the following evening providing a dosage of no more than 10 mg per day has been administered in the last 24 h.

### Intervention, randomisation and allocation

Treatment will be administered by ward staff orally in capsule form. Study product may also be administered via nasogastric or percutaneous endoscopic gastrostomy (PEG) tube. Each active capsule will contain 5 mg olanzapine and 137.5 mg Flocel (filler). Placebo capsules will contain 142.5 mg Flocel (filler). Dosage will be flexible and titrated as follows: (1) one capsule at night (5 mg olanzapine or matching placebo per day); (2) one capsule in the morning, one capsule at night (10 mg olanzapine or matching placebo per day); (3) one capsule in the morning, two capsules at night (15 mg olanzapine or matching placebo per day); and (4) two capsules in the morning, two capsules at night (20 mg olanzapine or matching placebo per day). Dosage may be escalated every 3 to 4 days. The decision to escalate dose will be made based on whether ABS score reduces between dose escalation days, the clinical appropriateness of dose escalation and whether that participant’s ABS score is in the agitated range (ABS > 21). Should a participant’s ABS score fall below the agitated range (ABS < 22) whilst in PTA, gradual dose reduction to a minimum dose of one capsule at night (5 mg olanzapine or matching placebo per day) may occur in consultation with the participant’s treating doctor and study psychiatrist. All dosage changes for each participant will be reported. Specific data to determine dose range in this setting is not available. We therefore chose this dose range based on Australian regulatory approval of olanzapine dosages of 5–20 mg to treat other neuropsychiatric conditions, such as schizophrenia and bipolar I disorder [46].

Randomisation to groups (29 participants, or 50% of the total sample of 58 participants per group) will be undertaken by an independent Epworth HealthCare accredited biostatistician employing permuted blocks of random length [47] in line with Consolidated Standards of Reporting Trials (CONSORT) 2010 guidelines [48], employing a procedure such as the ralloc procedure of Stata version 15 or higher. Block sizes will not be disclosed, to facilitate concealment. The randomisation schedule will be supplied by the independent biostatistician to a compounding pharmacist at an on-site compounding pharmacy located at Epworth HealthCare, who will prepare sequentially numbered, sealed opaque envelopes containing the group assignment. Allocation lists in the possession of the independent biostatistician and pharmacy will be stored securely in a password-protected file or locked cabinet.

Following eligibility confirmation, the participant will be enrolled into the trial by study staff and assigned the next available participant number. Randomisation will be requested of the on-site compounding pharmacy by study staff and the pharmacy will dispense treatment in line with that participant’s allocation. Thus, randomisation will be conducted without any influence of allocation awareness by participants, ward staff or investigators. Study product will be compounded as needed in batches. Once randomisation has taken place, a prescription for olanzapine containing the patient’s details including participant number will be signed by the participant’s treating physician and sent to the on-site pharmacy. The pharmacy will then bottle the treatment as appropriate, based on the randomisation schedule. This will be labelled with the participant’s details including study participant number and name. Study product dosage, and any escalation in dosage, will be reflected in the participant’s drug chart. Study treatment will be dispensed in quantities deemed appropriate by study staff, as treatment duration (i.e. PTA duration) is participant-dependent. Study product will be dispensed by the pharmacy and transported to the ward via usual Epworth HealthCare distribution methods or by study staff. Further prescriptions will be signed and sent to the pharmacy as needed.

Upon emerging from PTA as judged by a score of 12/12 on the WPTAS on three consecutive days, olanzapine will be tapered at a rate deemed appropriate by study doctors. Dose reduction or rechallenge may be undertaken in the setting of an adverse event (AE). Intervention may also be discontinued in the following circumstances: (1) following 6 months in PTA, (2) withdrawal of MTDM consent, (3) due to medical considerations or (4) escalation of agitation to an unmanageable level, resulting in physical aggression posing threat of harm to staff or property.

To maximise compliance, study staff will regularly check that treatment has been administered as prescribed by reviewing the participant’s medical chart. Study staff will also complete a treatment log, recording whether dosages have been administered, and dosage amount.

### Rescue medication

In the event that agitation levels escalate to an unmanageable level such that this results in physical aggression posing threat of harm to staff or property, participants may be administered a rescue dose of olanzapine. This decision has been made in the interest of participant retention and to represent such highly agitated patients in the participant pool. Multiple incidents of rescue medication use will prompt consideration of withdrawal from participation by the trial Data Safety Monitoring Committee (DSMC) in consultation with the patient’s treating doctor and study psychiatrist.

### Participant timeline

The participant timeline is displayed in Table 1 schedule of activities.

**Table 1:** Schedule of activities

Study phase	Screening/consent	Baseline/randomisation	Treatment period (PTA)	Post-PTA	Discharge	Three-month follow-up
ACD/ MTDM identification	X					
Trial introduction	X					
MTDM consent	X					
Screening	X					
Participant consent				X		
Randomisation		X				
Demographics^a^		X		X		
Medical record^b^		X	X		X	
Medical information^c^		X	X	X		
Blood test(s)^d^		X	X	X		
WPTAS		X	X			
ABS		X	X	X	X	
Simpson-Angus Scale		X	X			
Administer treatment			X			
Treatment log			X			
AE monitoring^e^			X			
RAVLT				X		
SDMT				X		
SF-12				X	X	X
FIM				X	X	
MPAI-4						X
PDHSUQ						X

^a^Age, gender, years of education, marital status, pre-injury employment/study status, drug and alcohol history, psychiatric history

^b^One or more of date and cause of injury, Glasgow Coma Scale, PTA duration, imaging results, surgical intervention, other injuries, Injury Severity Score, therapy hours and number of sessions, special nursing care, medication details, number of patients on ward

^c^Weight, BP, HR, WC

^d^Assessing fasting lipids, fasting glucose, HBA1C, creatinine

^e^Respiratory issues, lethargy, nausea, vomiting, difficulty swallowing, constipation, urinary retention, dizziness, seizures

#### Baseline—day 0

Following eligibility confirmation and randomisation, the following information will be gathered from the participant’s medical record: baseline WPTAS and ABS, demographic information (age, gender, years of education, marital status, pre-injury employment/study status, drug and alcohol history and psychiatric history) and medical information (date and cause of injury, Glasgow Coma Scale score, PTA duration, imaging results, surgical intervention, other injuries, injury severity score, therapy hours, special nursing care and medication details). Pre-treatment measures of weight, blood pressure (BP), heart rate (HR) and waist circumference (WC) will be conducted by nursing staff. Blood tests assessing fasting lipids, fasting glucose, haemoglobin A1c (HBA1C) and creatinine will be conducted. The Simpson-Angus Scale (SAS; [49]) will be completed by the patient’s treating physiotherapist, in order to assess the presence of extrapyramidal symptoms.

#### Treatment—day 0 until emergence from PTA (or 6 months maximum)

The treatment period will last from day of completion of baseline assessments until the patient is deemed to have emerged from PTA or until 6 months have elapsed. Emergence from PTA is defined as the first of three consecutive days on which a score of 12/12 on the WPTAS is achieved. Participants will be administered their randomised dose. They will also receive optimal environmental management as per guidelines [7]. WPTAS and ABS scores will be recorded by nursing staff or the patient’s clinical neuropsychologist. Need for dosage escalation and rescue medication use will be monitored by study staff. To describe concomitant care and inform health-economic evaluation, the number of patients on the ward, therapy hours and number of sessions and medication use including name and dose regimen will be recorded from the participant’s medical file.

AE monitoring will occur weekly. Clinically significant changes in these parameters will be recorded as AEs and a decision made by the treating doctor in consultation with a psychiatrist and the DSMC as to whether serious AEs necessitate withdrawal from the study.

#### Post-PTA—3 days following emergence from PTA (or 6 months)

Study staff will be informed by ABI ward staff when participants emerge from PTA and treatment will be titrated down as appropriate. Participant consent will be sought as soon as possible following emergence from PTA. Study staff will ask the participant to provide any demographic information unable to be gained from their medical record. Post-PTA measures of weight, BP, HR, WC and blood tests assessing fasting lipids, fasting glucose, HBA1C and creatinine will be conducted. The ABS will be completed daily for 2 weeks following emergence from PTA. Cognitive performance will be measured using the Rey Auditory Verbal Learning Test (RAVLT; [50]) and Oral Symbol Digit Modalities Test (SDMT; [51]). Functional independence will be measured using the Functional Independence Measure (FIM; [52]) and the 12-Item Short Form Health Survey Version 2 (SF-12; [53, 54]). These measures will be completed within 2 weeks of emergence from PTA. If participants remain in PTA at 6 months, treatment will be ceased and measures of weight, BP, HR, WC and blood tests will be administered at this time point. Remaining measures corresponding to the post-PTA time point will be administered upon the participant’s emergence from PTA.

#### Discharge

Study staff will be notified by ABI ward staff when the participant is ready to be discharged from Epworth HealthCare. Within 1 week prior to hospital discharge, any additional medical information not previously available in the participant’s medical record will be gathered, the ABS will be completed and functional independence will be measured using the FIM and the SF-12.

#### Three-month post-discharge follow-up

Participants and their MTDM will be contacted at 3 months post-discharge via telephone. Participants will complete the SF-12, their MTDM will complete the Mayo Portland Adaptability Inventory-4 (MPAI-4; [55]) and both parties will complete the Post-Discharge Health Service Utilisation Questionnaire (PDHSUQ). These measures will be completed within 3 weeks of the calculated follow-up date.

The participant’s MTDM may not be in a position to answer the questions posited in the MPAI-4 and PDHSUQ (for example, if they live in different countries or do not have a close relationship). In such cases, researchers will ask the participant to nominate another party who the researchers may contact to gather this information. Participants will be thanked for their participation and debriefed, including answering of any questions they might have regarding the study.

### Concomitant and post-trial care

All participants will receive environmental management designed to minimise agitation [7] including being located in a secure and quiet ward, avoiding restraint and overstimulation, allowing frequent rest and regulation of visitors to avoid overstimulation. Dedicated, consistent staff will optimise communication, reassurance and orientation. Participants will be permitted to engage in whatever other medical, rehabilitative or pharmacological therapy their medical team deems appropriate. Exceptions to this allowance include the use of medications for agitation (not including trial or rescue medication).

Following completion of the study, participants will continue to receive usual rehabilitation and other services as offered by Epworth HealthCare. Epworth HealthCare will also provide treatment for any drug-related AEs.

### Outcomes and measures

#### Primary outcome

To assess the efficacy of the atypical antipsychotic olanzapine in reducing agitation in patients in PTA following TBI over and above recommended environmental management, we will examine the difference between active and placebo groups’ mean daily score on the ABS during PTA. This outcome will include all daily ABS assessments throughout the treatment period. The ABS is a widely used measure of agitated behaviour in TBI [56–58]. Completion involves the observer rating the presence of 14 agitated behaviours based on the past 8-h period. Internal consistency is high, and construct validity and inter-rater reliability have been established [57–60]. Nurses and clinical neuropsychologists who complete the ABS at Epworth HealthCare are trained in its use.

#### Secondary outcomes

1. To examine the safety of olanzapine administration in PTA patients: Difference between olanzapine and placebo groups’ mean number of AEs (as captured via trial bloodwork, Adverse Events Questionnaire, Simpson-Angus Scale, measures of weight, BP, HR, WC) during PTA.
2. To determine whether olanzapine treatment reduces the number of clinically agitated days during PTA: Difference between olanzapine and placebo groups’ mean number of clinically agitated days (ABS score > 21) during PTA.
3. To determine whether olanzapine treatment alters level of agitation at hospital discharge: Difference between olanzapine and placebo groups’ mean ABS score at discharge, controlling for ABS score at treatment commencement.
4. To determine whether olanzapine treatment alters level of cognition in PTA: Difference between olanzapine and placebo groups’ mean levels of PTA as measured using the WPTAS [44, 45], controlling for WPTAS score at baseline. Mean scores will encompass daily assessments of the WPTAS throughout the treatment period, from first day of treatment until emergence from PTA (defined as the first of three consecutive days on which a score of 12 on the WPTAS is obtained). The WPTAS is commonly used in Australia to assess PTA. Seven items on the WPTAS assess orientation to time, person and place, and five assess anterograde memory (therapist face, name and three picture cards). Good construct validity and inter-rater reliability have been demonstrated [45, 61]. Nurses and clinical neuropsychologists who administer the WPTAS at Epworth HealthCare are trained in its use.
5. To determine whether olanzapine treatment alters PTA duration: Difference between olanzapine and placebo groups’ mean number of days in PTA (from date of injury until emergence from PTA based on three consecutive scores of 12 on the WPTAS).
6. To determine whether olanzapine treatment alters cognitive performance following PTA emergence:
  - Difference between olanzapine and placebo groups’ mean total words recalled over five trials on the RAVLT [50]. The RAVLT is a 15-word cognitive list learning task. This test is sensitive to memory impairments in TBI populations [62] and has good psychometric properties [63].
  - Difference between olanzapine and placebo groups’ mean number of items completed on the SDMT [51]. The SDMT is a test of processing speed, requiring transcription of numbers corresponding with symbols from a key over 90 s. Use of the oral version of this test will avoid effects of motor weakness on performance. The oral SDMT has excellent psychometric properties [64].
  - Difference between olanzapine and placebo groups’ mean number of errors on the SDMT.

Epworth HealthCare clinical neuropsychologists who will administer these measures are trained and experienced in their use.

7. To determine whether olanzapine treatment alters length of inpatient hospital stay: Difference between olanzapine and placebo groups’ mean length of inpatient hospital stay, measured in days from day of admission until day of discharge from inpatient care.
8. To determine whether olanzapine treatment alters functional independence at discharge and 3-month follow-up:
  - Difference between olanzapine and placebo groups’ mean scores on the FIM [52] at hospital discharge. The FIM is a measure of motor and cognitive functional status. It has good reliability [65] and is well validated in brain injury populations [66]. This measure will be completed by the participant’s treating occupational therapist, who is trained in its use.
  - Difference between olanzapine and placebo groups’ mean scores on Mental Component Summary (MCS) and Physical Component Summary (PCS) subscales of the SF-12 at hospital discharge. The SF-12 measures the patient’s view of their health-related quality of life. PCS and MCS scores are derived. The SF-12 is commonly used in TBI samples [67] and has excellent psychometric properties in the general population [53].
  - Difference between olanzapine and placebo groups’ mean scores on the MPAI-4 [55] at follow-up. The MPAI-4 assesses physical, cognitive, emotional, behavioural and social sequelae of TBI and obstacles to community integration on 3 subscales: Ability Index, Adjustment Index and Participation Index. It has strong psychometric properties [68], is used widely in TBI research and is more sensitive to disability after return to the community than the FIM [63]. The MPAI-4 will be completed via telephone interview with the study participant’s MTDM. Traumatic brain injury patients often present with issues with reduced self-awareness and memory. By having those knowledgeable about the patient’s functional independence complete this measure, problems with self-awareness and memory within this population are circumvented.
9. To evaluate the cost-effectiveness of olanzapine administration:
  - Total cost per patient to 3-month follow-up, calculated by combining standard unit costs [69] with patient-level data on health service utilisation to 3-month follow-up and estimates of productivity gains/losses. Health service utilisation will be estimated based on medical record indicated medication use, therapy hours, length of inpatient hospitalisation, special nursing care and PDHSUQ as completed by the patient and their MTDM at 3-month follow-up. Once again, data collection from the point of view of the MTDM at this time point aims to overcome difficulties associated with poor self-awareness and memory in TBI patients. The PDHSUQ has been designed for the purposes of this trial to assess participants’ post-discharge use of medications, allied health services, psychologists or counsellors, general practitioner visits, use of paid or unpaid carers and admissions to hospital. Productivity gains/losses will be estimated based on inpatient length of inpatient hospitalisation, FIM scores at discharge and MPAI-4 scores at 3-month follow-up.
  - Incremental cost of olanzapine and environmental management versus placebo and environmental management, calculated per patient to 3-month follow-up as the (adjusted) difference between olanzapine and placebo groups’ mean total cost per patient to 3-month follow-up.
  - Incremental cost per unit improvement on the ABS (mean daily ABS scores for olanzapine and placebo groups during PTA).
  - Incremental cost per point improvement on the FIM between PTA emergence and hospital discharge (mean scores for olanzapine and placebo groups).
  - Incremental cost per quality-adjusted life year gained (mean scores for olanzapine and placebo groups calculated using the SF-12 SF-6D utility index scores and derived using recently published Australian weights [70]).

### Blinding

The on-site compounding pharmacy, the independent Epworth HealthCare accredited biostatistician who is responsible for creation of the randomisation schedule and the DSMC will be unblinded to group allocation. Olanzapine and placebo capsules will be as visually identical as possible to facilitate comparability of blinded interventions. Participants, MTDMs, ward staff, study staff and all investigators involved in the study will be blinded to treatment allocation. Unblinding will occur following data collection, once data cleaning has been completed.

Emergency unblinding may occur in response to an AE, where knowledge of treatment is required in order to manage the participant’s condition. This will occur in partnership with the DSMC, who will monitor outcomes and be informed of serious AEs and withdrawals. Any intentional or unintentional breaking of the blind will be reported and explained.

### Retention

To maximise data collection at 3 months post-discharge, participants and MTDM will be reminded at discharge that they will be contacted in 3 months, and told the approximate date of this contact. If a participant or MTDM fails to respond to attempts at 3-month contact, study staff will make every effort to regain contact (via telephone, email and, if necessary, a letter to their last known mailing address). These contact attempts will be documented. Should the participant continue to be unreachable, he or she will be considered to be lost to follow-up. These participants will not be withdrawn from the study, and existing data will be analysed.

Those participants who are withdrawn from the study before the cessation of PTA will be debriefed upon PTA emergence, and informed consent for the use of previously collected data and continuing data collection on outcome measures will be sought in order to allow for intention-to-treat analysis. There will also be continued recording of symptoms as per AE follow-up, if applicable. Any incidents of non-retention and reason for this will be documented.

### Sample size

Simulation studies by Teare et al. [71] indicate that small studies (less than 50 participants, 25 per group) do not provide realistic estimates of results, indicating that group sizes should be at least 25, and that preferably a minimum of 35 participants per group be employed where possible.

Although it did not specifically include the ABS, a recent systematic review [41] reported standardised mean differences (i.e. Cohen’s *d* or the difference in group means divided by the pooled group standard deviation) in 2-h and 24-h post-administration levels of agitation between intramuscular olanzapine and placebo ranging from 0.36 to 1.93. In regard to other populations, a study of agitation levels in patients with schizophrenia following administration of intramuscular olanzapine or placebo found a standardised mean difference of 1.27. The minimal clinically important difference for improvement in irritable/aggression scale scores in TBI [68] is equal to a standardised mean difference of 0.50, the conventional Cohen medium effect size, with a large Cohen effect size being defined as a standardised mean difference of 0.80. Given the above prior findings, a large effect size would be expected. Using the Stata 15 power and sample-size procedure, we calculated the required sample size to detect a standardised mean difference/effect size of 0.80 (e.g. 0.80 of the standard deviation of 4.1 for mean daily ABS score at outcome reported in the pilot study of McKay et al. [26], *n* = 125) with a statistical power of 0.80 and a 2-tailed alpha of 0.05. Results indicated a required sample size of 26 patients per group, for a total of 52 patients.

A sample size of 26 patients per group, or 52 in total, exceeds the lower threshold of 50 patients identified by Teare et al. [71] and is clinically practical. Assuming 10% attrition, 29 patients (26/(1–0.10) = 28.9, conservatively rounded to 29) per group would be required, for a total of 58 patients, in order to complete the study with 52 patients.

### Statistical methods

#### Analysis of primary and secondary endpoints

Descriptive statistics will be reported using frequencies and percentages for categorical data. Percentages will be calculated based upon the number of patients for whom data are available. Continuous or dimensional variables will be summarised using means and standard deviations, with medians and interquartile ranges (difference between 25th and 75th percentiles) reported for potentially skewed data such as SF-12 PCS and MCS scores.

For the analysis of the primary endpoint, difference in mean daily ABS scores during PTA between olanzapine and control groups, a mixed model/multilevel regression framework [72], taking into account duration of PTA, will be employed. Although a difference between group means corresponding to a large effect size (0.80 of the pooled group standard deviation) is expected, a clinically significant improvement is defined as a difference in group means of the mean daily ABS scores within PTA equal to at least half a pooled group standard deviation difference, the minimal clinically important difference for improvement in irritable/aggression scale scores in TBI [68]. In regard to secondary endpoints, regression methods appropriate to the type of outcome variable will be employed. Comparison of the two groups on dimensional secondary endpoints such as ABS at discharge and WPTAS scores will be analysed using linear regression or median quantile regression. Median quantile regression, based upon the median and so less affected by very high or very low scores, will be employed for possibly skewed variables, such as SF-12v2 MCS and PCS scores, that may not meet the assumptions of linear regression [73].

Secondary endpoints involving count data including number of days in PTA, number of clinically agitated days, length of stay in hospital, agitation scores on discharge, number of “treatment failures” and number of AEs and other measures of treatment safety will be analysed using regression models for count data (Poisson or negative binomial regression as appropriate; [74]).

All statistical tests will be two-tailed, with alpha or statistical significance level set to *p* < 0.05. 95% confidence intervals will be reported throughout. The number of missing observations will be reported. Analyses will assume intention to treat, with all patients analysed as randomised.

If imputation of missing data is required, the method of imputation will depend on the amount of missing data, its characteristics and whether it is the covariates and/or outcome variables that are missing [75]. Following suggested practice, complete case analysis will be reported along with imputation. Multiple imputation will be undertaken separately for each group, and a sensitivity analysis for the assumptions (i.e. missing completely at random) performed [75]. Full details of the imputation procedure will be reported [76]. Statistical analysis will be conducted by a professional Epworth HealthCare or Epworth HealthCare accredited biostatistician, blinded to group assignment, employing a standard statistical package such as Stata 15 or higher. A detailed Statistical Analysis Plan will be prepared by the biostatistician(s).

#### Cost-effectiveness analysis

In line with the main analysis, the primary outcome for the economic evaluation will be mean score on the ABS during PTA. Secondary outcomes for the economic evaluation will be FIM at hospital discharge and quality-adjusted life years to 3-month follow-up calculated using SF12-based Six-Dimensional Health State Short Form (SF6D) scores derived using recently published Australian weights [70]. Treatment effects with respect to the ABS and FIM will be estimated as for the main analysis. Treatment effects with respect to the SF6D index score and total quality-adjusted life years to 3-month follow-up will be estimated using one-part generalised linear models, controlling for SF6D index scores at pre-treatment and specifying appropriate variance and link functions [77].

Patient-level data on total cost during inpatient rehabilitation admission will be extracted from patient medical records to capture medication use, therapy hours, length of stay and special nursing care. Post-discharge health service utilisation will be estimated from patient/MTDM self-report (at 3-month follow-up) regarding use of primary care, prescription and over-the-counter medications, allied health care, community care and paid and unpaid home-help as reflected on the PDHSUQ. Productivity gains/losses will be estimated based on length of inpatient hospitalisation, FIM scores at discharge and MPAI-4 scores at 3-month follow-up. Given the likely structure of our data and the advice of Buntin and Zaslavsky [78], treatment effects with respect to total cost will be estimated using one-part generalised linear models with gamma variance function and a log link (rather than transformed ordinary least squares or two-part models), controlling for patient characteristics at pre-treatment.

Results will be expressed as (i) cost per point improvement on the ABS, (ii) cost per point improvement on the FIM, and (iii) cost per quality-adjusted life year gained. We will summarise sampling error and parameter uncertainty using the bootstrap acceptability method to calculate confidence intervals and generate cost-effectiveness acceptability curves [77].

### Harms

A DSMC has been established. The DSMC is independent of the study team and consists of a psychiatrist (chair), neuropsychiatrist and biostatistician. The DSMC will monitor outcomes and be informed of serious AEs and withdrawals. The DSMC will advise study staff on (1) dose reduction, rechallenge or withdrawal in response to AEs, in consultation with the treating doctors; (2) withdrawal of participants due to escalation of agitation to an unmanageable level, resulting in physical aggression posing threat of harm to staff or property, in consultation with study psychiatrist; and (3) necessity of emergency unblinding in response to an AE. No further DSMC charter exists or is currently planned to be developed. The DSMC will not engage in interim analyses. Should a decision need to be made regarding early trial termination, this decision will be made jointly between study principal investigators and the DSMC.

Potential AEs will be monitored in the following ways:

1. Medical information—Weekly measures of weight, BP, HR and WC will be conducted by nursing staff at baseline, weekly throughout the treatment period and following emergence from PTA. Plans for the collection, laboratory evaluation and storage of blood samples are detailed in the provided (Additional file 1).

2. Blood tests—Blood tests assessing fasting lipids, fasting glucose, HBA1C and creatinine will occur at several time points: at baseline, upon the participant’s first score of 12 on the WPTAS (an early indicator of emergence from PTA), monthly throughout the treatment period and following emergence from PTA. Plans for the collection, laboratory evaluation and storage of blood samples are detailed in the provided Appendix: Biological Specimens.

3. The SAS [49]—This scale will be completed by the participant’s treating physiotherapist at baseline and weekly throughout the treatment period. The SAS is a rating scale for extrapyramidal system disturbance widely used to assess side effects related to antipsychotic drug use. It has well-established psychometric properties [49].

4. Adverse Events Questionnaire—This questionnaire will prompt reporting of AEs expected due to olanzapine use. This questionnaire will be completed weekly throughout the treatment period by the participant’s treating physician in partnership with study staff. Symptoms assessed will include respiratory issues, lethargy, nausea, vomiting, difficulty swallowing, constipation, urinary retention, dizziness and seizures. Information to be collected includes event description, start date, severity, relatedness to treatment, action with investigational product, expectedness, seriousness, outcome, end date and classification.

Clinically significant changes in these parameters will be recorded as AEs. Adverse events will be tabulated and tracked in a password-protected electronic file. All AEs will be followed to adequate resolution or stabilisation, including those that persist beyond the treatment period. If AEs continue beyond hospital discharge, follow-up will occur via telephone.

The DSMC will be informed of all serious AEs. A decision will be made by the treating doctor in consultation with a study psychiatrist and the DSMC as to whether withdrawal from the study, dose reduction or rechallenge is necessary.

Notification and reporting of AEs will be carried out according to the Australian regulatory, ethics committee and International Conference on Harmonisation Good Clinical Practice guidelines.

### Auditing

The investigators will permit study-related monitoring, institutional review board/institutional ethics committee review and regulatory inspection(s) and provide direct access to source data/documents.

### Data management, confidentiality and access

Participant case report forms and source documents will be stored securely at the Monash Epworth Rehabilitation Research Centre (MERRC). Data will be entered electronically at MERRC from original study materials into a password-protected file for analysis. Data transmitted electronically will be password protected.

To maintain anonymity, participants will be allocated a participant number. All raw and analysed data from tests and questionnaires will be labelled with that participant’s identifying number and will not contain information that could enable identification of individual participants. Any pre-existing identifying information contained within copies of original data collection materials (e.g. ABS, WPTAS, medical record) will be blacked out. A password-protected digital file containing each participant’s name, contact information and numerical identifier will be kept, separate from the study data. Only individuals authorised by the principal investigators will have access to study files. Data will be retained for 15 years from the date of publication of results.

All investigators will have access to study data. Data in de-identified electronic form may be made available to other researchers for the purpose of peer review. Data in this form may also be used for the purposes of further analysis by individuals authorised by the principal investigators.

### Dissemination policy

There are no publication restrictions associated with this study. Results will be published in international peer-reviewed journals and presented at academic conferences, regardless of the magnitude or direction of effect. Every attempt will be made to reduce to an absolute minimum the interval between the completion of data collection and the publication of study results. We expect to take approximately 6 months to compile the final results paper for an appropriate journal. Study results will also be published in the trial registry. Authorship criteria published by the International Committee of Medical Journal Editors [79] will be followed when submitting manuscripts for publication. The investigators do not intend to make the anonymised dataset or statistical code for generating results publicly available.

Upon the analysis of data, participants will be sent a letter informing them of study results, should they elect for this to occur in the Participant Information and Continuing Consent Form.

## Discussion

Agitated behaviour is distressing to patients, staff and families and is associated with poorer patient outcomes. Clinicians may employ environmental or pharmacological strategies when managing agitation in PTA patients. However, no quality evidence supports the use of any strategy for the management of agitation. Pharmacological intervention is common, and the use of antipsychotics amongst the most prevalent choice for treatment. Though preferred over their typical counterparts, the efficacy, safety and long-term outcomes associated with the use of atypical antipsychotics within this population are yet to be established. This trial aims to examine the efficacy of the atypical antipsychotic olanzapine in reducing agitation in PTA patients following TBI, over and above environmental management.

The major challenge associated with this trial has thus far been recruitment. This is thought to be the result of apprehension of MTDMs to consent to the patient’s participation in the trial, as well as difficulty fulfilling inclusion and exclusion criteria in a medically complex population. We will continue to explore possible avenues to mitigate these issues. One possible drawback of this trial is that the inclusion of olanzapine as rescue medication does not allow for a strict division between the treatment regime of placebo and olanzapine groups. However, this provision is expected to allow for the retention of highly agitated participants who would otherwise remain unrepresented in this sample, and those who would possibly be excluded following a single incident of escalation in agitation. This provision has also been made to protect the safety and promote the engagement of medical staff involved in the day-to-day care of these patients. The number of rescue doses administered to participants will be compared between groups, providing valuable insight into in whom and under what circumstances rescue medication was required.

The results of this trial will represent a major step forward in understanding the efficacy, safety and long-term outcomes associated with olanzapine use in this unique population. The results of this trial will inform the care of these patients, whose current standard of care is based predominantly on low-level evidence and physician’s clinical experience.

### Trial status

Recruitment for this project began in June 2019 and is expected to be completed by June 2022. The current recruitment status is 5. The protocol version at the time of submission is version 1.4, 21 October 2019.

## Supplementary information

**Additional file 1: Appendix.** Biological Specimens.

## Data Availability

Not applicable

## Abbreviations

ABI: Acquired brain injury
ABS: Agitated Behaviour Scale
ACD: Advance Care Directive
AE: Adverse event
BP: Blood pressure
CONSORT: Consolidated Standards of Reporting Trials
DSMC: Data safety monitoring committee
FIM: Functional Independence Measure
HBA1C: Haemoglobin A1c
HR: Heart rate
MCS: Mental component summary
MERRC: Monash Epworth Rehabilitation Research Centre
MPAI-4: Mayo Portland Adaptability Inventory-4
MTDM: Medical Treatment Decision Maker
PCS: Physical component summary
PDHSUQ: Post-Discharge Health Service Utilisation Questionnaire
PEG: Percutaneous endoscopic gastrostomy
PTA: Post-traumatic amnesia
RAVLT: Rey Auditory Verbal Learning Test
SAS: Simpson-Angus Scale
SDMT: Symbol Digit Modalities Test
SF6D: Six-Dimensional Health State Short Form
SF-12: 12-Item Short Form Health Survey Version 2
TBI: Traumatic brain injury
WC: Waist circumference
WPTAS: Westmead Post Traumatic Amnesia Scale
