# Supplementary material for: Use of olanzapine to treat agitation in traumatic brain injury: study protocol for a randomised controlled trial
Source: Trials. 2020 Jul 20;21:662. doi: 10.1186/s13063-020-04553-2 (PMC7370410; doi:10.1186/s13063-020-04553-2)
Supplement: Supplementary file 1 — Additional file 1: Appendix. Biological Specimens. [file 13063_2020_4553_MOESM1_ESM.docx]

**Use of olanzapine to treat agitation in traumatic brain injury: Study protocol for a randomised controlled trial.**

**Appendix: Biological Specimens (SPIRIT Checklist Item 33)**

Trial participants undergo blood tests assessing fasting lipids, fasting glucose, HBA1C and creatinine. These analyses occur at several time points: At baseline, upon the participant’s first score of 12 on the Westmead Post Traumatic Amnesia Scale, monthly throughout the treatment period, and following emergence from post-traumatic amnesia.

Blood will be drawn at these time points by Melbourne Pathology staff, who have been contracted to perform blood draws and analysis. Participants will fast for 8-15 hours prior to having their blood drawn. These samples will be collected, transported, stored and analysed according to strict Melbourne Pathology protocols. Blood samples will be stored securely on-site at the Melbourne Pathology laboratory undertaking analysis. Following analysis, samples will be destroyed at the earliest possible convenience of the laboratory. There will be no future use of blood samples in ancillary studies.

To protect participant anonymity, blood samples will be labelled with participant number, date of birth and sex. No further identifying information will be included on the blood test request form, tube labelling or results.

Results of blood sample analysis will be imparted to the research team via the Melbourne Pathology secure online portal and fax.
